# Supplementary material for: End Sequence Analysis Toolkit (ESAT) expands the extractable information from single-cell RNA-seq data
Source: Genome Res. 2016 Oct;26(10):1397–410. doi: 10.1101/gr.207902.116 (PMC5052061; doi:10.1101/gr.207902.116)
Supplement: Supplemental Material [file supp_gr.207902.116_Supp_Materials.tar.gz › ESAT-esat_v0.1/doc/html/classumms_1_1core_1_1alignment_1_1_single_end_alignment_1_1_wrap_sam_record.html]

ESAT: umms.core.alignment.SingleEndAlignment.WrapSamRecord Class Reference


ESAT


- Main Page
- Related Pages
- Packages
- Classes
- Files

- Class List
- Class Index
- Class Hierarchy
- Class Members

All Classes Namespaces Files Functions Variables Enumerator Pages

- umms
- core
- alignment
- SingleEndAlignment
- WrapSamRecord

Public Member Functions |
List of all members

umms.core.alignment.SingleEndAlignment.WrapSamRecord Class Reference

Inheritance diagram for umms.core.alignment.SingleEndAlignment.WrapSamRecord:

[legend]

Collaboration diagram for umms.core.alignment.SingleEndAlignment.WrapSamRecord:

[legend]

|  |  |
| --- | --- |
| Public Member Functions | |
|  | WrapSamRecord (SAMRecord record) |
|  | |
| int | getMappingQuality () |
|  | |
| boolean | getDuplicateReadFlag () |
|  | |
| void | setDuplicateReadFlag (boolean duplicateFlag) |
|  | |
| String | getReadString () |
|  | |

## Detailed Description

This class is a wrapper for the SAMRecord class so that this class can be correctly serialized

Author
:   skadri

## Constructor & Destructor Documentation

|  |  |  |  |  |  |
| --- | --- | --- | --- | --- | --- |
| umms.core.alignment.SingleEndAlignment.WrapSamRecord.WrapSamRecord | ( | SAMRecord | *record* | ) |  |

## Member Function Documentation

|  |  |  |  |  |
| --- | --- | --- | --- | --- |
| boolean umms.core.alignment.SingleEndAlignment.WrapSamRecord.getDuplicateReadFlag | ( |  | ) |  |

|  |  |  |  |  |
| --- | --- | --- | --- | --- |
| int umms.core.alignment.SingleEndAlignment.WrapSamRecord.getMappingQuality | ( |  | ) |  |

|  |  |  |  |  |
| --- | --- | --- | --- | --- |
| String umms.core.alignment.SingleEndAlignment.WrapSamRecord.getReadString | ( |  | ) |  |

|  |  |  |  |  |  |
| --- | --- | --- | --- | --- | --- |
| void umms.core.alignment.SingleEndAlignment.WrapSamRecord.setDuplicateReadFlag | ( | boolean | *duplicateFlag* | ) |  |

---

The documentation for this class was generated from the following file:

- java/umms/core/alignment/SingleEndAlignment.java


---

Generated on Fri Aug 15 2014 15:11:07 for ESAT by  

 1.8.7
